# The Westernmost Record of the Scyphomedusa *Cassiopea andromeda* (Forskål, 1775) in the Mediterranean: Marine Citizen Science Contributions to Invasive Species Detection and Monitoring

**DOI:** 10.1007/s00267-025-02289-w

**Published:** 2025-09-30

**Authors:** Macarena Marambio, Maria Pascual-Torner, Uxue Tilves, Alejandra Pérez, Ainara Ballesteros, Josep-Maria Gili

**Affiliations:** 1 Department of Marine Biology and Oceanography, Institut de Ciències del Mar (ICM-CSIC), Barcelona, Spain; 2 Aquatours Almería Aventuras Submarinas, Club Náutico Aguadulce Local bajo, Almería, Spain; 3 IMEDMAR-UCV – Institute of Environment and Marine Science Research, Universidad Católica de Valencia SVM, Calp, Alicante Spain

**Keywords:** NIS, Lessepsian invasion, Climate change, Participatory science, Thermophilic jellyfish

## Abstract

The Mediterranean Sea, although a biodiversity hotspot, is one of the most affected seas by non-indigenous species (NIS). This problem is worsened by rising sea temperatures due to climate change, which promotes the spread of thermophilic species. Among the NIS scyphozoan jellyfish species recorded in the Mediterranean, *Cassiopea andromeda* – commonly known as the “upside-down jellyfish”– is a notable example. *Observadores* del Mar (*OdM*) is the leading platform for marine citizen science in Spain and works towards ocean conservation and health. It is a well-established tool for generating knowledge in marine research and has successfully provided early warning of NIS reports in the Mediterranean, while also serving as an effective network for the monitoring of NIS and other indicators. Three reports of *C. andromeda* from Almeria, southern Spain, have been reported in *OdM* and thanks to the involvement of its community, 12 samples were collected for phylogenetic analysis and monitoring was done for 15 months in the study area. The results confirmed the first record of *C. andromeda* in Spanish Mediterranean waters representing the westernmost record in the basin. Monitoring also suggests the species establishment in the area. This study contributes to the knowledge of *C. andromeda* invasiveness and highlights the importance of marine citizen science in the detection and monitoring of NIS. It also underscores the collaboration and commitment already established between scientists and citizens, which will allow further progress in the fields of biological invasions, management, and policy.

## Introduction

The Mediterranean Sea, listed as one of the main hotspots in the world, is home to more than 17,000 marine species, accounting for 7% of the world’s total species (Coll et al. 2010). However, this biodiversity is changing because of various human-mediated impacts, such as species introduction, direct or indirect (Coll et al. 2010; Stock et al. 2018). In particular, the presence of non-indigenous species (NIS), also called exotic, non-native, or alien species, has been used as a biodiversity loss indicator (Katsanevakis et al. 2014), and invasive alien species are considered one of the major threats to marine biodiversity (Roy et al. 2019; Pyšek et al. 2020).

The Mediterranean Sea is one of the most affected seas by biological invasions (Zenetos et al. 2010). Recent studies have listed over 950 alien species introduced into the Mediterranean (Zenetos et al. 2020), a fact that has been favored since the opening of the Suez Canal in 1869 (Galil 2012). The Suez Canal, indeed, is the main entry point for NIS into the Mediterranean (Katsanevakis et al. 2013; Galil et al. 2018), a phenomenon known as “Lessepsian migration”. For jellyfish, scyphozoans in particular, there are records of 18 species in the Mediterranean (reviewed in Badreddine and Bitar 2020), and at least 5 of them are Lessepsian migrants (Galil et al. 1990, 2013, 2017); *Rhopilema nomadica* Galil et al. 1990; *Phyllorhiza punctata* von Lendenfeld 1884; *Cotylorhiza erythraea* Stiasny 1920; *Marivagia stellata* Galil et al. 2017; and *Cassiopea andromeda* (Forskål 1775).

**Fig. 1:**
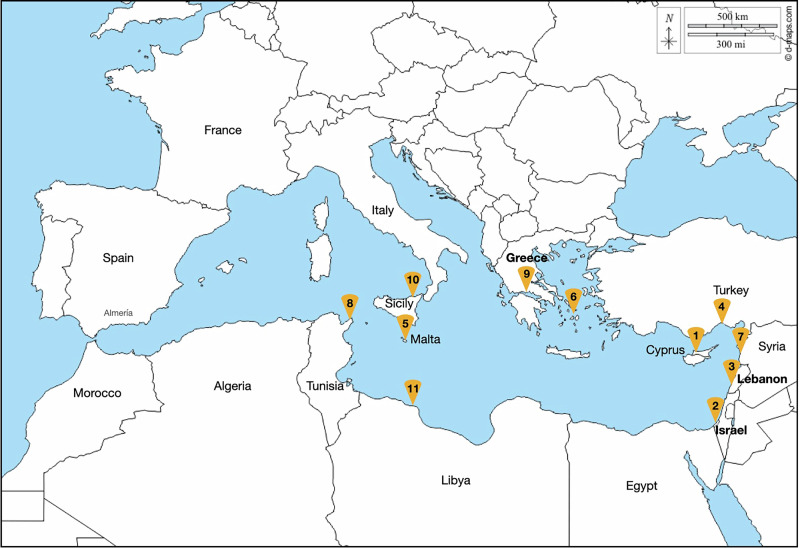
First records of *Cassiopea andromeda* in the Mediterranean Sea after the Suez Canal opening. **1**: Maas (1903); **2-3**: Galil et al. (1990); **4**: Çevik et al. (2006); **5**: Schembri et al. (2010); **6**: Zenetos et al. (2011); 7: Siokou et al. (2013); **8**: Amor et al. (2015); **9**: Yokes et al. (2018); **10**: Cillari et al. (2018); 11: Crocetta et al. (2021). The map was obtained and modified from www.d-maps.com

*Cassiopea andromeda*, native to the Red Sea and the Indo Pacific (Mariotini and Pane 2010), was the first Lessepsian jellyfish species described after the opening of the Suez Canal. It was first sighted in Cyprus in 1903 (Mass 1903), and since then, it has been reported (at least punctually or repeatedly observed) in almost the entire eastern basin (Goy et al. 1988; Spanier 1989; Çevik et al. 2006; Schembri et al. 2010; Zenetos et al. 2011; Siokou et al. 2013; Yokes et al. 2018; Servello et al. 2019; Crocetta et al. 2021) (Fig. 1). Regarding the western basin, the presence of *C. andromeda* has been documented on rare occasions, and the only records come from northern Tunisia (Amor et al. 2015) and Sicily (Cillari et al. 2018; Maggio et al. 2019). Later, Cassiop*ea* sp. was reported in Mar Menor (Murcia, Spain) in 2017 (Rubio 2017); however, it was an occasional grey-literature report of a single small individual without species identification.

Within the genus *Cassiopea* (order Rhizostomeae), which contains several species (e.g., *C. mayeri, C. frondosa, C. ornata, C. xamachana, C. andromeda*), *C. andromeda* is the only species reported in the Mediterranean Sea. Most reports in this area identify *C. andromeda* by morphology without confirming it through molecular barcoding (Cillari et al. 2022; Deidun et al. 2018; Çevik et al. 2006), except for that from Palermo, Sicily (Maggio et al. 2019). However, Cassi*opea* is considered a cryptic species, therefore identification based on physical characters such as the color or pattern of spots at the exumbrella, number of rhopalia, shape and number of mouth appendages, length of oral arms, or arrangement of canals in the umbrella, are not entirely reliable and rigorous, as they can be highly variable between individuals of the same species (Jarms and Morandini 2019). For this reason, the use of molecular tools such as 16S ribosomal RNA (16S) and mitochondrial cytochrome c oxidase subunit I (COI) gene identity is especially relevant in any study involving this genus (Muffett and Miglietta 2023; Holland et al. 2004). In addition, NIS such as *C. andromeda* can also be detected through environmental DNA (eDNA), a powerful and cost-effective method capable of identifying species even when they are not yet conspicuous. For instance, *C. andromeda* was detected via eDNA along the northern, eastern, and southern coasts of Sicily, during periods when medusae were not visually observed (Aglieri et al. 2023).

*C. andromeda* is commonly known as the “upside down jellyfish” because it lies on its umbrella surface to expose to the light the symbiotic dinoflagellates of the family Symbiodiniaceae (De Domenico et al. 2025) present in their oral arms to facilitate photosynthesis (Lampert et al. 2011). This species typically inhabits warm and well-illuminated shallow waters such as mangroves and seagrass beds and areas with muddy or sandy bottoms. Recent studies have presented records of *C. andromeda* in harbors, such as the case of Malta (Schembri et al. 2010) and Augusta and Palermo in Sicily (De Rinaldis et al. 2021; Cillari et al. 2018, 2022).

*C. andromeda* jellyfish is considered an invasive species (Katsanevakis 2011), and like every invasive species, it may have an ecological impact, as it can proliferate rapidly, forming large blooms within a short time frame (Zenetos et al. 2011; Deidun et al. 2018). Additionally, it possesses certain characteristics that could facilitate its spread and establishment, particularly its high tolerance to thermal stress (Banha et al. 2020), being able to survive at 13 °C (Deidun et al. 2018) but also at 36 °C (Çevik et al. 2006). Moreover, increasing temperatures due to global change enhance growth and sexual reproduction making it a species capable of proliferating in the currently warming conditions (Mammone et al. 2023). On the other hand, it may also have a socio-economic impact affecting tourism and human health as it is considered a mid-stinging species; therefore, prevention and mitigation measures must be taken where it is present, as well as the development of species-specific stinging protocols (Ballesteros et al. 2023).

With the rapid expansion of NIS in the Mediterranean, the use of early detection and warning tools is essential to prevent significant ecological and/or socio-economic impacts caused by the establishment of these species in marine ecosystems. Marine citizen science, volunteers participating in marine research (Thiel et al. 2014), is expanding and gaining great value (Earp and Liconti 2020). Evidence shows that it is a robust tool for providing scientific data for biodiversity conservation (McKinley et al. 2016), improving scientific monitoring at large scales (Bonney et al. 2009; Dickinson et al. 2010), and contributing to research on biological invasions by acting as a tool for early detection of NIS (Delaney et al. 2008; Crall et al. 2010; Giovos et al. 2019; Tiralongo et al. 2020; Encarnação et al. 2021). Another recent and very useful early detection method is the environmental DNA metabarcoding (eDNA) survey, that can be used to monitor NIS in some specific areas (Aglieri et al. 2023; Ye et al. 2024).

*Observadores* *del Mar* (*OdM*; www.observadoresdelmar.es), the marine citizen science platform of reference in Spain, is focused on marine conservation, answering questions to improve the understanding of marine ecosystems and working beyond ocean health. Currently, the platform houses 15 projects addressing five relevant topics: marine biodiversity, vulnerable species, marine impacts, climate change, and exotic and invasive species. *OdM* has managed to establish a very committed community in its 13 years of experience made up of several actors: scientific expert teams, stakeholders, collaborating organizations, the *Sentinel Observatory* network, and the large community of volunteers. The *Sentinel Observatory* (*SO*) network was created in 2016, currently comprising over 20 diving centers and clubs, associations, and other entities. The objective of this *SO* network is to establish a more systematic data collection and monitoring by expanding even more the spatio-temporal range, therefore, acting as an early alert and becoming a functional network. The *SO* network goes one step further in their commitment with *OdM* by systematically monitoring one or more projects of the platform through increased sampling, project-specific dives or research into a particular data. They report on a recurring basis and *OdM* provides them with specific training and a more constant and dedicated exchange of information.

*OdM*’s commitment and dedication is not only to the *SO* network, but to the community in general, with the aim of providing with sufficient tools to ensure the correct collection and veracity of data, to analyze and obtain results and answers to the research questions. For this reason, different tools have been developed and made available, including identification guides, a large photo database accessible to participating citizens, adapted standardized protocols, online and in-person training, as well as validation by an expert scientific team, resulting in high scientific value and an excellent-quality database (Figuerola-Ferrando et al. 2023; Coppari et al. 2024).

As for jellyfish research, various initiatives have demonstrated the effectiveness of citizen science (reviewed in Marambio et al. 2021), some of which provide valuable information about the ecology, spatio-temporal distribution, and the socio-economic impact in certain coastal areas (De Donno et al. 2014; Kienberger and Prieto 2017; Marambio et al. 2021; Tirelli et al. 2021). In *OdM*, one of the most successful projects is “*Jellyfish Alert”* (Marambio et al. 2023), which has been part of the platform since its creation in 2012 and currently has a very active community. The project is focused on data collection related to the presence and absence of gelatinous zooplankton organisms, including “true jellyfish” (scyphozoans), native and non-native, hydrozoans, ctenophores and salps. Data of one or few individuals and blooms are commonly registered, and records come mainly from the Mediterranean but also from around the world. In the years that the project has been active, a database of more than 2500 observations has been created including valuable records of the species present mainly on the Spanish coast. Recently, a protocol for monitoring climate change indicator species has been included in the project to collect data more systematically and help understand the effects of rising sea temperatures on the population and reproductive cycles of some common Mediterranean jellyfish species.

This study aims to report the first phylogenetically confirmed record of the scyphomedusa *C. andromeda* (Forskal, 1775) in Spanish waters and the westernmost record of the species in the Mediterranean Sea, which has formed large aggregations of a self-sustained reproductive population and has remained established in the last year, even expanding its distribution in the waters of the Aguadulce Marina in Almeria, southern Spain. Furthermore, this work emphasizes the importance of marine citizen science initiatives such as *OdM* and the involvement of its community in reporting the presence of the species, collecting samples for further scientific analyses, and monitoring the population with a temporarily systematic approach.

## Materials and Methods

### Study Area

**Fig. 2:**
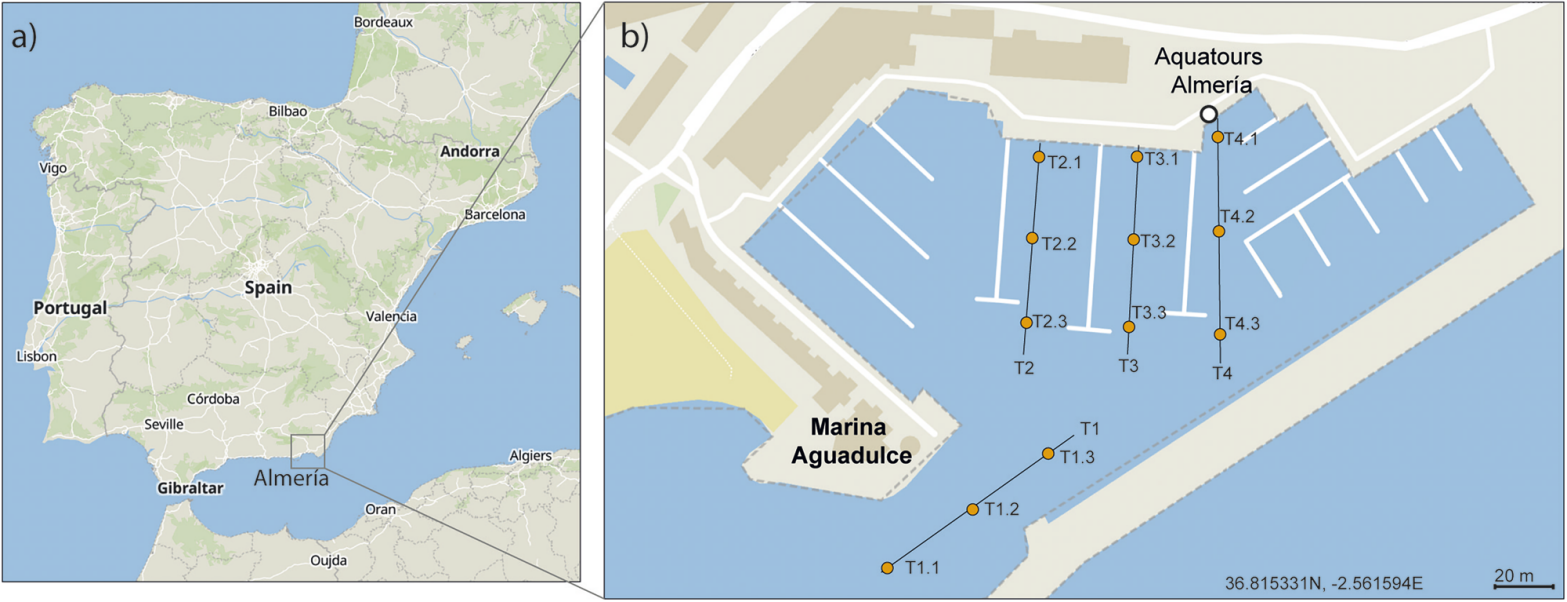
**a** Map of Spain highlighting the coast of Almeria as the study area; **b** Zoom into the Marina Aguadulce study area, highlighting the sampling protocol used for individual collection. The transects (T1, T2, T3, and T4) represent the sampling paths where *C. andromeda* individuals were found in 2023. Orange circles indicate collection sites

The Marina Aguadulce is located at 36° 48.51’ N and 2° 33.42’ W, in the town of Roquetas de Mar, 8 km from Almería, province of the Autonomous Community of Andalusia in Spain (Fig. 2). It is mainly a touristic port with a total area of 170,462 m^2^ and more than 750 moorings available. The sides of the different channels of the marina are formed by rocks, but the central part is made of silt (~15 cm deep) and has an approximate width of 5–8 m depending on the channel.

### Record, Sample Collection and Monitoring

**Fig. 3:**
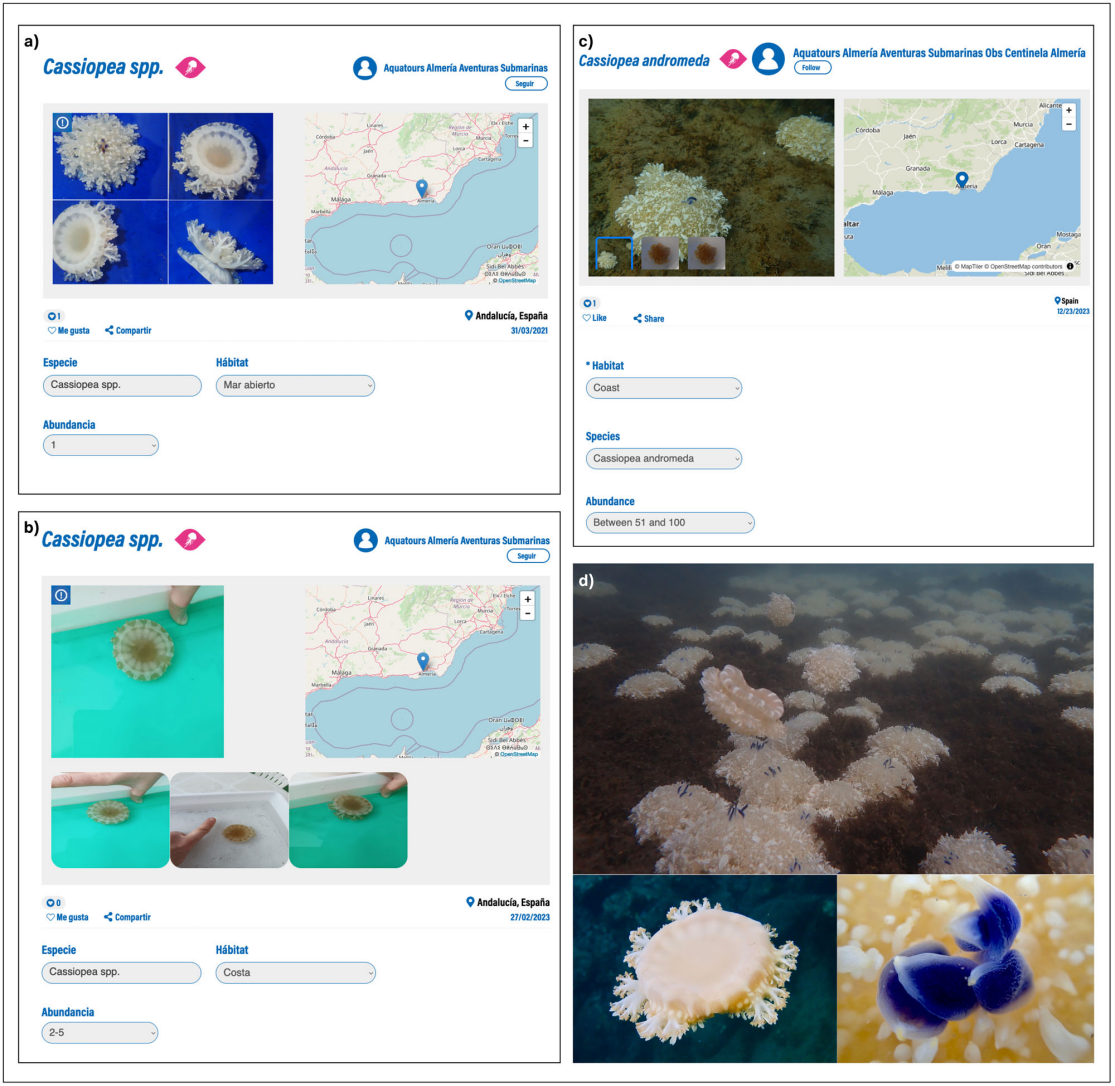
**a** Screenshot of *Cassiopea* spp. observation uploaded to *OdM* in March 2021; **b** Screenshot of *Cassiopea* spp. observation uploaded to *OdM* in February 2023; **c** Screenshot of *C andromeda* observation uploaded to *OdM* in December 2023; **d** Photographs of the population and individuals registered in the Marina Aguadulce in Andalucia in December 2023. The complete observation can be accessed at https://www.observadoresdelmar.es/Observations/3/23385

Three observations (March 2021, February 2023 and December 2023) of *Cassiopea* individuals were registered in the *Jellyfish Alert* project in the *OdM* platform by one *SO* of the network. The last record, from December 2023, corresponded to an aggregation of considerable abundance (i.e. more than 10 individuals/m^2^; Fig. 3d), therefore the expert scientific team considered it relevant to carry out a phylogenetic analysis as it is a cryptic NIS. For this purpose, twelve *Cassiopea* individuals of ~5 cm umbrella diameter were hand-collected by scuba divers at various locations (Fig. 2) within the study area on February 11 and 12, 2024. Temperature and salinity were recorded in each sampling point. The rationale for collecting twelve individuals distributed along the Marina Aguadulce was to account for the possibility of more than one species coexisting, as other studies have detected for *Cassiopea* (Muffett and Miglietta 2023). After collection, the specimens were preserved in 96% ethanol at room temperature and analyzed three days later.

When first observed in December 2023, population density was estimated in one of the seven channels (4 m wide × 50 m long) (corresponding to T4 in Fig. 2), using 1 × 1 m quadrants. The umbrella diameter of 52 specimens was measured to estimate a rough size range. Additionally, monitoring was carried out for 15 months, from December 2023 until February 2025, and consisted of observing the presence and absence of *Cassiopea* in each of the seven channels that comprise the marina, as well as in the entrance channel, to assess its establishment and adaptation in the study area.

### DNA Sequencing

DNA was extracted from each of the twelve *Cassiopea* specimens from a small portion of the umbrella margin following the standard phenol‒chloroform protocol. The quantity of DNA was assessed using Nanodrop, and its quality was checked via agarose 1% gel electrophoresis. Mitochondrial cytochrome c oxidase subunit I (COI) and 16S ribosomal RNA (16S) were amplified using primers ‘LCO1490-JJ2’ (5′-CHACHACWAAYCAYAARGAYATYGG-3′) and ‘HCO2198-JJ2’ (5′-ANACTTCNGGRTGNCCAAARAATCA-3′) for COI and ‘C&B1’ (5′-TCGACTGTTTACCAAAAACATAGC-3′) and ‘C&B2’ (5′-ACGGAATGAACTCAAATCATGTAAG-3′) for 16S, as described by Gamero-Mora et al. (2022). The polymerase chain reaction (PCR) was performed under the same conditions for both COI and 16S: 5 min at 94 °C for initial denaturation, followed by 35 cycles of amplification (denaturation at 94 °C for 15 s, annealing at 53 °C for 15 s and elongation at 72 °C for 45 s) and a final extension for 5 min at 72 °C. The PCR products were validated through 1% gel electrophoresis, purified by 1:5 dilution and Sanger sequenced at Stab Vida S. A.

### Data Analysis

The 16S and COI sequences from the 12 individuals in Almeria Harbor collected in this study were aligned using the ‘msa’ function (from the ‘msa’ package) with other *Cassiopea* sequences available in the GenBank database: *C. andromeda, C. xamachana, C. mayeri, C. culionensis*, and *C. frondosa*, as well as with the outgroup species *Mastigias papua* and *Phyllorhiza punctata* (Table 1). The alignments were trimmed to 546 bp for 16S and 514 bp for COI using ‘msaTrim’ (from the ‘microseq’ package). Phylogenetic analyses were conducted separately for 16S and COI using maximum likelihood as the optimality criterion for each. The optimal substitution model was selected via ModelFinder, choosing the model with the lowest Akaike information criterion (AIC) score: TIM2 + F + I + G4 for 16S and TIM2 + F + I for COI (Kalyaanamoorthy et al. 2017). Each optimal model was bootstrapped 1000 times to generate the final consensus phylogenetic tree with branch support values (Hoang et al. 2018). The phylogenetic analyses were performed via IQ-TREE multicore (ver. 2.3.2). The consensus trees were visualized in Rstudio (RStudio Team 2020) using the ‘ggtree’ package, which was previously rooted in the outgroup species *M. papua* with the ‘root’ function (from the ‘ape’ package).

**Table 1:** Sequences of the mitochondrial ribosomal gene 16S rRNA and the mitochondrial protein-encoding gene cytochrome c oxidase I (COI) were used for phylogenetic analysis

Species	Location	16S GenBank accession	COI GenBank accession	Source
*Cassiopea andromeda*	El Ghardaqa, Egypt	–	AY319458	Holland et al. (2004)
*Cassiopea andromeda*	Baja California Sur, Isla San Jose, Mexico	KY610611	KY610551	Gómez Daglio and Dawson (2017)
*Cassiopea andromeda*	Cudjoe Key, Florida, USA	OP503932	OP503345	Muffett and Miglietta (2023)
*Cassiopea andromeda*	Key Largo, Florida, USA	OP503939	OP503367	Muffett and Miglietta (2023)
*Cassiopea andromeda*	Almería, Spain	**CSA331484**	**–**	This study
*Cassiopea andromeda*	Almería, Spain	**CSA331485**	**CSA331497**	This study
*Cassiopea andromeda*	Almería, Spain	**CSA331486**	**CSA331498**	This study
*Cassiopea andromeda*	Almería, Spain	**CSA331487**	**CSA331499**	This study
*Cassiopea andromeda*	Almería, Spain	**CSA331488**	**–**	This study
*Cassiopea andromeda*	Almería, Spain	**CSA331489**	**CSA496001**	This study
*Cassiopea andromeda*	Almería, Spain	**CSA331490**	**–**	This study
*Cassiopea andromeda*	Almería, Spain	**CSA331491**	**CSA496003**	This study
*Cassiopea andromeda*	Almería, Spain	**CSA331492**	**CSA496004**	This study
*Cassiopea andromeda*	Almería, Spain	**CSA331493**	**CSA496005**	This study
*Cassiopea andromeda*	Almería, Spain	**CSA331494**	**–**	This study
*Cassiopea andromeda*	Almería, Spain	**CSA331495**	**CSA496007**	This study
*Cassiopea andromeda*	Palermo, Sicily	–	Ca2_CaCOIF^b^	Maggio et al. (2019)
*Cassiopea andromeda*	Palermo, Sicily	–	Ca1_CaCOIF^b^	Maggio et al. (2019)
*Cassiopea andromeda*	Palermo, Sicily	–	Ca4_CaCOIF^b^	Maggio et al. (2019)
*Cassiopea culionensis*	Lapu-Lapu, City of Cebu, Philippines	MW164869	MW160913	Gamero-Mora et al. (2022)
*Cassiopea culionensis*	Lapu-Lapu, City of Cebu, Philippines	MW164879	MW160923	Gamero-Mora et al. (2022)
*Cassiopea culionensis*	Lapu-Lapu, City of Cebu, Philippines	MW164886	MW160930	Gamero-Mora et al. (2022)
*Cassiopea frondosa*	West Key, Florida, USA	KY610617	AY319467	Holland et al. (2004), Gómez Daglio and Dawson (2017)
*Cassiopea mayeri*	Ryukyu Islands, Okinawa, Japan	MW164859	MW160931	Gamero-Mora et al. (2022)
*Cassiopea mayeri*	Lapu-Lapu, City of Cebu, Philippines	MW164863	MW160934	Gamero-Mora et al. (2022)
*Cassiopea mayeri*	Lapu-Lapu, City of Cebu, Philippines	MW164864	MW160935	Gamero-Mora et al. (2022)
*Cassiopea mayeri*	Calatagan, Luzon Island, Philippines	MW164865	MW160936	Gamero-Mora et al. (2022)
*Cassiopea mayeri*	Calatagan, Luzon Island, Philippines	MW164866	MW160937	Gamero-Mora et al. (2022)
*Cassiopea ornata*	Kakaban, Kalimantan, Indonesia	AB720918	AY319472	Holland et al. (2004) and Gamero-Mora et al. (2022)
*Cassiopea ornata*	Guam, USA	OL721669	OL799293	Anthony et al. (2022)
*Cassiopea xamacana*^a^	Bahia Delfines, Bocas del Toro, Panama	KY610613	KY610558	Gómez Daglio and Dawson (2017)
*Cassiopea xamacana*^a^	Bahia Delfines, Bocas del Toro, Panama	KY610614	KY610559	Gómez Daglio and Dawson (2017)
*Cassiopea xamachana*	Tavernier, Florida, USA	OP503922	OP503334	Muffett and Miglietta (2023)
*Cassiopea xamachana*	Lobster Walk, Monroe County, Florida, USA	OP503929	OP503341	Muffett and Miglietta (2023)
*Mastigias papua*	Risong Cove, Palau	KU901021	KU901397	Swift et al. (2016)
*Phyllorhiza punctata*	Gulf of California, Mexico	MT902932	MT904380	Rosales-Catalán et al. (2021)

GenBank accession numbers of sequences in bold were obtained in this study

Species with ‘^a^’ were reported as *C. frondosa* in the sequence publication but fall into the *C. xamachana* clade, which is supported by Muffett and Miglietta (2023)

^b^Indicates that the sequences are not available in GenBank and were given by the authors

## Results

After genetic analysis of the twelve collected specimens, the results revealed that they corresponded to the species *Cassiopea andromeda*, becoming the first phylogenetically confirmed record of this species in Spain and the westernmost record of the Mediterranean basin. The observation was validated in the *Jellyfish Alert* project of the *OdM* platform as a confirmed record of *C. andromeda* species (Fig. 3c). The other two previous observations, from March 2021 and February 2023, were validated morphologically and only at the genus level because no phylogenetic analysis was performed (Fig. 3a, b).

During the sampling in February 2024, the temperature was 14.33 ± 0.12 °C and the salinity 37.58 ± 0.08. The aggregations of *C. andromeda* were observed in various channels, mainly in the central silt area, covering the length and width of the channels at an approximate depth of 4–5 m. Some of them were also observed over the meadows of another invasive species, the *Rugulopteryx okamurae* algae, which has also colonized areas of the marina. No individuals were observed over the rocky lateral areas.

Individuals were characterized by a brownish color with whitish and blue rounded and flattened vesicles (Fig. 3d). During the monitoring carried out in December 2023, the density of the population was estimated to be 80–100 individuals/m^2^ and the umbrella diameter ranged from 4 to 30 cm.

**Fig. 4:**
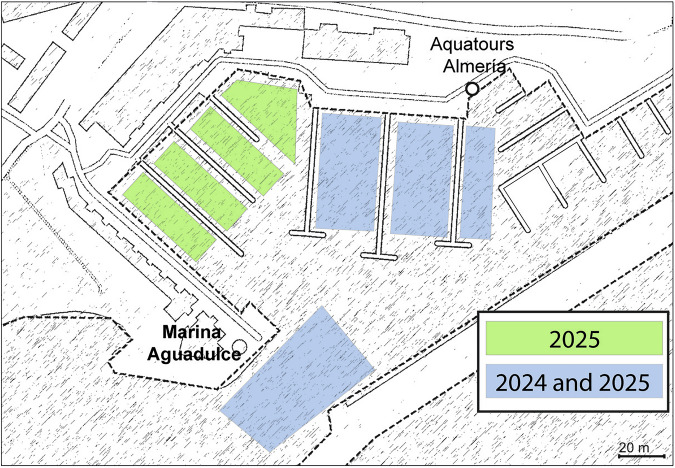
Representation of the population’s expansion inside the Marina Aguadulce, by indicating in green areas where individuals appeared after 1-year monitoring

The monitoring conducted over 15 months indicated that the population has survived all seasons and has even expanded its distribution, having already colonized all the marina channels by 2025 (Fig. 4). Moreover, different stages and sizes of individuals have been observed, indicating active reproduction in the study area.

**Fig. 5:**
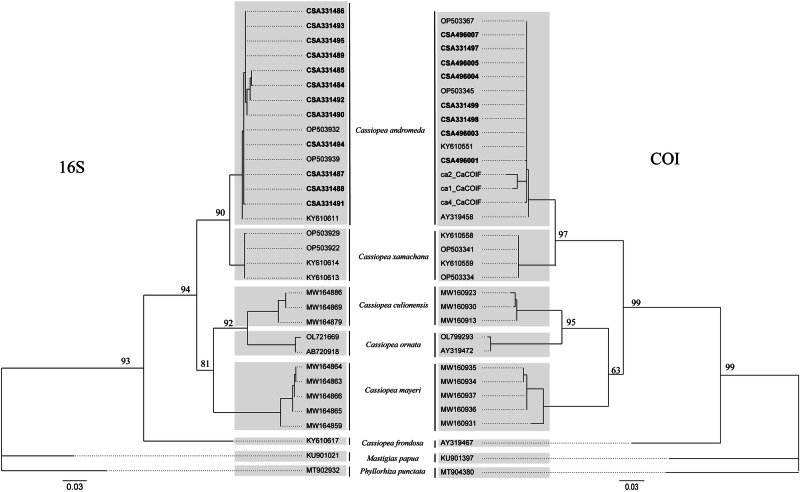
Phylogenetic tree illustrating the evolutionary relationships among different *Cassiopea* species. Maximum likelihood consensus tree with bootstrap support for (left) the mitochondrial ribosomal gene 16S rRNA and (right) the mitochondrial protein-encoding gene cytochrome c oxidase I (COI) DNA. Names in bold indicate sequences generated in the present study. Gray squares group sequences that belong to the same clade. Scale bars indicate evolutionary distance. See Table 1 for information about all the sequences used

Phylogenetic analyses of the 12 sequences of *Cassiopea* from Almeria, Spain, combined with 24 additional sequences (most of which are available in the GenBank database), confirmed that *Cassiopea* specimens from Almeria clearly belong to *C. andromeda*. Four COI sequences (from sampling points T1.1, T2.2, T3.1, and T4.1 in Fig. 2), although matching the species, exhibited several nucleotide variations that led to unexpected stop codons and failed the NCBI quality check. Therefore, we assumed these sequences had Sanger sequencing errors and excluded them from the analyses to include only high-quality sequences. All specimens were clearly grouped within the clade of *C. andromeda*, which also includes specimens from Egypt, Florida Keys, and Sicily. At the same time, the phylogenetic tree revealed the presence of other clades, such as *C. xamachana*, which was closest to *C. andromeda*, along with *C. culionensis, C. ornata, C. mayeri*, and *C. frondosa*. The bootstrap values for most nodes of the tree were greater than 80%, indicating that the consensus maximum likelihood tree was highly reliable (Fig. 5).

Sequences from Almería showed 100% nucleotide identity with the two sequences from Florida, except for one COI sequence that differed by a single nucleotide and four 16S sequences that differed by five nucleotides. However, the molecular distance, measured by the Kimura 2-parameter (K80), among the sequenced individuals remained very low (0.2 ± 0.2% for 16S and 0.0 ± 0.1% for COI). These values did not differ from those of *C. andromeda* from Egypt, Florida, and Sicily (16S: 0.2 ± 0.2% and COI: 0.2 ± 0.3%). The intraspecific variation in *C. andromeda* (16S: 0.2 ± 0.2% and COI: 0.2 ± 0.3%) was much smaller than the interspecific variation (16S: 11.2 ± 5.1% and COI: 14.3 ± 6.0%). Accordingly, all these groups presented high genetic distance from the outgroup species *M. papua* and *P. punctata* (16S: 21.0 ± 1.5% and COI: 25.1 ± 1.9%) (Supplementary Information file).

## Discussion

According to Zenetos et al. (2020), almost 1000 marine NIS have been introduced into the Mediterranean. Although the number of alien species in the Mediterranean varies between regions (Zenetos et al. 2012), the majority occur in the Eastern sub-region. The Sicily Channel plays an important role in this distribution pattern since it has been traditionally considered a biogeographical barrier that prevents the spread of these species, restricting them to the eastern basin (e.g., Quignard and Tomasini 2000). Nevertheless, some species have crossed the Sicily channel and have been found in the western region. This is the case for some Lessepsian scyphomedusae species, such as our target species *Cassiopea andromeda* (Morandini et al. 2017; Aljbour et al. 2017; Maggio et al. 2019), and others, such as *Rhopilema nomadica* (Balistreri et al. 2017) and *Phyllorhiza punctata* (Deidun et al. 2017).

*C. andromeda* is one of the 18 species of jellyfish reported as NIS in the Mediterranean. Its records, although not all phylogenetically confirmed, are diverse. Until now, the species had not been confirmed in Mediterranean Spanish waters, and its westernmost record was from Italy (Maggio et al. 2019). In this work, we report the first phylogenetically confirmed record of the NIS *C. andromeda* in Spanish waters and the westernmost record of this species in the Mediterranean Sea. The analysis suggests that the population in this location consists solely of *C. andromeda* (Fig. 5), excluding any coexistence with *C. xamachana*, as described in other studies (Muffett and Miglietta 2023). Overall, the bootstrap values (an indicator of the confidence in the placement of a particular clade within a phylogenetic tree) were between 80% and 99%, with few values lower than 70%, in accordance with other phylogenetic studies of *Cassiopea* species (Muffett and Miglietta 2023; Gamero-Mora et al. 2022; Arai et al. 2017). When comparing the COI and 16S trees, the former had higher bootstrap values, except for the node between the clades *C. ornata/C. culionensis* and *C. mayeri* (63%). However, the bootstrap value of the analogous node in the 16S tree was greater (81%). Therefore, the phylogenetic results in this study are highly reliable.

The phylogenetic tree, which uses either 16S or COI markers, indicates that each *Cassiopea* species diverged through distinct evolutionary paths, in accordance with other phylogenetic studies of *Cassiopea* (Gamero-Mora et al. 2022; Muffett and Miglietta 2023). These trees show that *C. frondosa* is distinct from the other species, indicating early divergence. Interestingly, this is the only *Cassiopea* species that can be unequivocally identified by morphology (it has a different number of rhopalia) (Morandini et al. 2017). Later in evolution, there was a divergence into two distinct clades: the “*C. andromeda/C. xamachana*” and “*C. mayeri/C. ornata/C. culionensis*” clades, where *C. mayeri* diverged from the latter. Finally, *C. andromeda*-*C. xamachana*, and *C. ornata*-*C. culionensis* diverged from their most recent common ancestors, indicating recent evolution, and suggesting potential cryptic species within these groups (Fig. 5).

Crypticity is common in Scyphozoa and can lead to species misidentification when it is detected only by morphology (Moura et al. 2022; Holland et al. 2004; Dawson 2003). In the context of invasive species, crypticity complicates their detection and management, hampering the prediction and control of their impacts (Jarić et al. 2019). In that sense, when a species has been sighted in a new location, barcoding identification — alone or combined with morphological description — is indispensable to ensure correct identification and management. There is a large difference between naming a study species and labeling it with DNA barcodes (such as 16S or COI markers), with the latter being much more precise. In addition, if the generated DNA barcodes are stored in public databases, they may be available for future phylogenetic studies. For example, our phylogenetic tree revealed that *Cassiopea* from Panama, which Gómez Daglio and Dawson 2017 identified as *C. frondosa*, corresponds to *C. xamachana*, which is in accordance with the findings of Muffett and Miglietta (2023). This fact, together with other examples of reidentification of *Cassiopea* species (Gamero-Mora et al. 2022), highlights how DNA barcoding allows species identification to be adapted in future studies, considering that taxonomy can change over time.

The introduction pathway of *C. andromeda* into Spanish waters is unknown. As an epibenthic scyphozoan, this jellyfish has a very limited movement capability, and most of the translocations reported in this species are more likely to be related to maritime transportation rather than natural transport by currents or by its own displacement (Holland et al. 2004; Schembri et al. 2010). We hypothesize that *C. andromeda* from this study, possibly the polyp stage, could have arrived by vessel transportation and probably as biofouling, since the study area is a marina and does not have ballast water loads (i.e., ambient water loaded into ballast tanks of commercial vessels). This hypothesis agrees with the case of Turkey, where the authors also suggested that polyps may have arrived in ships as biofouling (Çevik et al. 2006) while the pelagic stages in ballast water (Özgür and Öztürk 2008). In any case, independent of the arrival pathway, the current environmental conditions in the study area are certainly suitable for the species to develop and establish large populations, as has been observed in the last year after its first detection. The presence of this species in harbors and marinas has also been described in other areas, such as Malta, Sicily and Turkey (Schembri et al. 2010; Cillari et al. 2018; Çardak et al. 2011, respectively). Harbors have been described as an ideal region for NIS introduction because of high maritime traffic (Ferrario et al. 2017) and, in the case of *C. andromeda* and its congeneric, it has been demonstrated that human-impacted coastal habitats may enhance its ability to sustain populations and contribute to its establishment (Çevik et al. 2006; Thé et al. 2023; Stoner et al. 2011).

Following the history of invasive species in the Mediterranean, several authors have suggested that finding Lessepsian species in the western basin may be considered an indicator of the warming trend of the Mediterranean Sea (Boero et al. 2009; Daly Yahia et al. 2013). Water warming facilitates the natural spread of tropical and subtropical species, enabling them to expand their distribution range (Lasram et al. 2008; Parravicini et al. 2015). In this context, the Mediterranean Sea stands out as one of the most significant and susceptible regions to climate change (Giorgi 2006; Lionello et al. 2012), experiencing a warming rate per decade that exceeds the global average by more than threefold. These climate change conditions, and the resulting increase in sea temperature, may support the distribution and establishment of thermophilic species such as *C. andromeda* (Çevik et al. 2006), which is considered to enhance its physiological response to global warming (Aljbour et al. 2017, 2019; Banha et al. 2020; Mammone et al. 2023), and whose thermal tolerance could promote an increase in the population and expansion of its geographic distribution range (Aljbour et al. 2019).

*C. andromeda* records in the Mediterranean are from semi-enclosed eutrophic shallow waters with low hydrodynamics (Maggio et al. 2019). This is the case for the harbors mentioned above and for nature reserves (Malta, Deidun et al. 2018), marine protected areas (Tunisia, Amor et al. 2015), the cooling water drainage channel of a factory (Turkey, Çevik et al. 2006) or lagoons (Turkey, Özgür and Öztürk 2008). These shallow areas, although quite stressful (e.g., high temperatures/irradiation and potential extreme salinity changes), have been demonstrated to be suitable for their establishment, probably because of the high tolerance of this jellyfish to environmental variation (Morandini et al. 2017; Mammone et al. 2021). In previous works, it has been described at different temperatures ranging from 14.1–17.6 °C in Palermo (Maggio et al. 2019), 13.36–14.49 °C in Malta (Deidun et al. 2018), and 29–36 °C in Turkey (Çevik et al. 2006; Özgür and Öztürk 2008). In this study, *C. andromeda* was first detected in winter (December‒February) when the water temperature was ~14.2 °C and has been monitored and observed throughout the year following the yearly temperature range. The high abundances (80‒100 individuals/m^2^) recorded in the present study, are much higher compared with other studies where the maximum abundances described were 30‒40 individuals/m^2^ (Niggl and Wild 2010), especially considering that it is very likely that the density is underestimated since according to the information reported by the *SO* during the monitoring reporting, the jellyfish were one on top of the other forming layers that did not allow counting all the individuals in each quadrant.

Marine citizen science, as a growing opportunity for marine research (Sandahl and Tøttrup 2020; Earp and Liconti 2020; Changeux et al. 2020; García-Soto et al. 2021), has been reported in previous studies as a highly valuable tool for increasing knowledge about jellyfish distribution (Johansen et al. 2021; Marambio et al. 2021; Tirelli et al. 2021; Edelist et al. 2022; Dobson et al. 2023; Terenzini et al. 2023). Additionally, it provides essential data for establishing and/or improving preventive programs to mitigate jellyfish impacts in some coastal areas. In recent years, another growing area of citizen science is the reporting of NIS, therefore considered a useful tool for expanding the scale of data collection, for early detection and for monitoring exotic and invasive species (Delaney et al. 2008; Crall et al. 2010; Mannino and Balistreri 2018; Giovos et al. 2019; Tiralongo et al. 2020; Pocock et al. 2024). All this represents a clear benefit in expanding exotic and invasive species knowledge, and in their monitoring, management, and related policy development (Groom et al. 2019; Pyšek et al. 2020; Price-Jones et al. 2022).

Detecting NIS as early as possible, along with monitoring and research, is essential for determining the ecological and socio-economic impacts that their presence and establishment could have on invaded areas (Giovos et al. 2019; Pocock et al. 2024). In these instances, marine citizen science requires significant involvement from volunteers, as participation goes beyond mere observation reporting. Therefore, it is essential that platforms and initiatives have a track record and are well established, with strong community engagement. In this sense, *OdM* has a large and highly engaged community of volunteers from different sectors and a robust network of Sentinel Observatories (*SO*). Aquatours Almeria, the diving center that reported the presence of *C. andromeda*, has been part of this *SO* network since the beginning. In fact, 75% of the *SO* network consists of diving centers or clubs, which represents a good opportunity for marine citizen science, as divers are considered one of the most committed user groups, according to previous studies (Martin et al. 2016; Lucrezi et al. 2018). Moreover, *OdM* has demonstrated its consistency in effectively contributing to the early detection of NIS, expanding knowledge and contributing to decision-making related to marine conservation (Azzurro et al. 2013, 2020; Castejón-Silvo et al. 2023; Figuerola-Ferrando et al. 2023).

The *OdM* platform, through its specific project *Jellyfish Alert*, provides the necessary identification clues and expert support to recognize jellyfish species easily under good conditions. However, in some cases phylogenetic analysis is required for a correct identification, especially for cryptic species such as *Cassiopea* individuals. When further analysis is needed, the close collaboration with the *OdM*’s *SO* enables the collection of samples as they immediately receive a protocol from the scientific team with instructions for sample collection, ultimately allowing for species confirmation. Furthermore, as a *SO* of the platform’s network, they regularly conduct structured monitoring at the same location during their year-round dives. This has allowed them to track the species for more than 12 months since its detection, and they have been able to observe and report on its reproduction and expansion in the colonized area. This will allow us to get valuable information to assess the impact of the species over time (Pocock et al. 2024). Moreover, with this confirmed record, the message can be expanded to the public and encourage attention to this species. It will also contribute to understanding the importance and impact of invasive species on marine ecosystems and contribute to adaptive management strategies within a citizen science approach (Giovos et al. 2019; Pocock et al. 2024).

## Conclusions

The detection of NIS is highly relevant to the ecology of ecosystems and the conservation of the marine environment. The case of *C. andromeda* is of particular interest because it can be easily transported as biofouling and/or ballast water, and when it arrives in a new area, it can easily adapt to different environmental conditions, being highly thermotolerant. These characteristics, together with rising sea temperatures due to climate change, make almost any point in the Mediterranean a suitable place for this species, which, in addition, can spread rapidly, affecting local populations. This study contributes to the knowledge of the NIS *C. andromeda* in the Mediterranean, presenting the first phylogenetically confirmed record in Spanish waters and the westernmost record in the basin, as well as the contribution to public DNA databases. On the other hand, marine citizen science has proven useful and, if well implemented, is a powerful tool that allows the expansion of spatial-temporal marine research, improves ecological understanding, and contributes to ocean literacy-enhancing knowledge. In the case of NIS, it has an important value as a detection tool that has been used in various taxonomic groups, including jellyfish. For instance, the present study demonstrates the relevance of the marine citizen science platform *OdM*, as it plays a fundamental role in the detection, sampling and monitoring of this species through its engaged community. The potential of marine citizen science in reporting the presence of certain species and acting as a warning tool is unquestionable, as the advantage of having a large and engaged community makes it a highly cost-effective tool. This collaboration between scientists and citizens is translated into advances in marine research, management and even policy. With more than 12 years of experience, *OdM* has demonstrated the commitment of its community, the importance of providing training and standardized protocols, and the quality of the data. Furthermore, it implements all the recommendations for the establishment and successful development of a marine citizen science platform, and its contributions thus far confirm its success.

## Supplementary information

Supplementary information

## Data Availability

DNA sequences were submitted to GenBank (accession numbers: PQ154578–PQ154589 for 16S sequences and PV533755–PV533762 for COI sequences).
